# Experimental studies addressing the longevity of *Bacillus subtilis* spores – The first data from a 500-year experiment

**DOI:** 10.1371/journal.pone.0208425

**Published:** 2018-12-04

**Authors:** Nikea Ulrich, Katja Nagler, Michael Laue, Charles S. Cockell, Peter Setlow, Ralf Moeller

**Affiliations:** 1 Space Microbiology Research Group, Radiation Biology Department, Institute of Aerospace Medicine, German Aerospace Center (DLR e.V.), Cologne (Koeln), Germany; 2 MPG Research Group CATs (Complex Adaptive Traits), Max Planck Institute for Terrestrial Microbiology, Heidelberg, Germany; 3 Advanced Light and Electron Microscopy (ZBS 4), Robert Koch Institute, Berlin, Germany; 4 School of Physics and Astronomy, UK Center for Astrobiology, University of Edinburgh, Edinburgh, United Kingdom; 5 Department of Molecular Biology and Biophysics, University of Connecticut Health Center, Farmington, Connecticut, United States of America; Beijing Forestry University, CHINA

## Abstract

The ability to form endospores allows certain Gram-positive bacteria (e.g. *Bacillus subtilis*) to challenge the limits of microbial resistance and survival. Thus, *B*. *subtilis* is able to tolerate many environmental extremes by transitioning into a dormant state as spores, allowing survival under otherwise unfavorable conditions. Despite thorough study of spore resistance to external stresses, precisely how long *B*. *subtilis* spores can lie dormant while remaining viable, a period that potentially far exceeds the human lifespan; is not known although convincing examples of long term spore survival have been recorded. In this study, we report the first data from a 500-year microbial experiment, which started in 2014 and will finish in 2514. A set of vials containing a defined concentration of desiccated *B*. *subtilis* spores is opened and tested for viability every two years for the first 24 years and then every 25 years until experiment completion. Desiccated baseline spore samples were also exposed to environmental stresses, including X-rays, 254 nm UV-C, 10% H_2_O_2_, dry heat (120°C) and wet heat (100°C) to investigate how desiccated spores respond to harsh environmental conditions after long periods of storage. Data from the first 2 years of storage show no significant decrease in spore viability. Additionally, spores of *B*. *subtilis* were subjected to various short-term storage experiments, revealing that space-like vacuum and high NaCl concentration negatively affected spore viability.

## Introduction

Microorganisms are virtually ubiquitous on Earth, capable of not only tolerating but adapting to nearly any environmental extreme. Discoveries on the vast distribution of microbial diversity have continuously challenged fundamental questions such as ‘what are the limitations of life?’ [1,2]. Our capacity to explore these questions and begin to understand the mechanisms of microbial survival are continuously improving [3,4]. One microbial survival strategy that has been the subject of thorough microbiological investigation is sporulation. It allows certain microbes including members of the *Bacillus* and *Clostridium* genera to form dormant, multi-layered endospores in response to nutrient depletion. In their inactive state, spores monitor their surrounding environment so that if conditions become favorable, they can break dormancy and resume vegetative cell growth [5]. Due to the applied importance of bacterial spores in the medical field [6–9], the food industry [10,11], and extraterrestrial environments [12–14], there has been extensive research on spore-forming bacteria.

Bacterial spores are able to endure a variety of prolonged external stresses such as desiccation, freezing, elevated temperatures in dry or wet conditions, a slew of toxic chemicals, high pressures, as well as UV and γ-radiation [10]. The extreme resistance of a spore is largely due to its multi-layer structure, consisting of (from out- to inside) the proteinaceous coat, the peptidoglycan cortex, the germ cell wall and the inner membrane, all of which surround the spore core that contains the DNA and other biomolecules. The spore coat constitutes the initial barrier to potentially harmful molecules, while the compressed inner membrane blocks the entry of many small DNA-damaging chemicals due to its low permeability [15]. The spore core’s properties also contribute to resistance to external stress agents, as it has a low water content, a high concentration of dipicolinic acid (DPA), and its DNA is saturated with α/β*-*type small acid-soluble spore proteins (SASPs) [16–18]. Additionally, spores contain efficient mechanisms for repair of DNA damage during revival, helping them to combat accumulated damage during the spores’ dormancy [19]. These characteristics allow spores to remain viable and outlast the majority of other organisms [20]. In fact, it has been posited that bacterial spores are among the most resistant life forms [10] and could be one of the longest-living cellular structures [21]. There are controversial reports of spores discovered in the guts of fossilized bees located in 25-million year-old amber [22], in ancient soils and aquatic sediments [23,24], and even in a 250-million year old primary salt crystal from the subterranean Salado Formation located near Carlsbad, NM [25]. Studies have also indicated that pathogenic spores (i.e. *Bacillus anthracis*) could potentially remain viable for extended periods of time [26–28].

In order to address current questions of spore resistance and longevity, spores of *Bacillus subtilis* represent a well-documented model system [20,29]. Thus, *B*. *subtilis* spores have been extensively explored as decontamination indicators in industrial settings [30–32], as well as for resistance to certain stress agents such as radiation [33], heat [34] and high salinity [35]. However, while we know that *B*. *subtilis* spores can remain in their dormant state for many years [10], the true longevity and limits of viable *B*. *subtilis* spores are not well understood. Further, to our knowledge, no systematic studies of spore desiccation resistance have been performed for an extended timescale. Studies of this nature also aid our understanding of what cellular components are most prone to degradation, presenting the opportunity to engineer microbes with greater capability to survive long-term stresses with applications to drug storage and storage during space exploration.

The 500-Year Microbial Experiment [36,37] is designed to address the following fundamental questions. (1) How long can a lifeform (e.g. *B*. *subtilis* spores) survive storage in extreme desiccation? (2) How are spores’ revival kinetics affected by this long-term storage? (3) What mathematical function describes the rate of spore death over long periods? The opportunity to carry out this experiment over a timescale more conducive with spore lifetimes might help us to begin answering these questions. Here, we report the first data from what will ultimately comprise a 500-year study, and as such, begin a record. By continuing to discover the limits of life, we may further understand how life has existed for over 3.5 billion years on Earth and the possibility of life existing elsewhere.

## Materials and methods

### Spore production and purification

All experiments were carried out with *Bacillus subtilis trpC*2 strain 168 (DSM 402) originally obtained from the German Collection of Microorganisms and Cell Cultures GmbH (DSMZ, Braunschweig, Germany). Spores were prepared by cultivation in double-strength liquid Schaeffer sporulation medium [38] with vigorous aeration at 37°C for 72 hours. Harvested spores were purified by washing with sterile water repeatedly followed by lysozyme and DNase I treatment for the removal of vegetative cells. Further, a heat inactivation step at 80°C for 10 min was performed to inactivate any remaining vegetative or germinating spores, and the spores were subsequently washed with sterile water and checked for purity by phase-contrast microscopy. Spore preparations were free (>99%) of vegetative cells, germinated spores and cell debris.

### Storage experiment preparation

**Fig 1:**
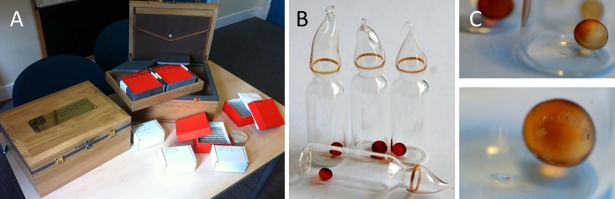
Experimental design. (A) The 500-Year Microbiology Experiment and its components. (B) Glass vials containing 100 μL of *B*. *subtilis* spore stock solution (10^6^ CFU/mL) and then dried down on silicon bead desiccators before being sealed as described in Methods. (C) Silica beads to maintain desiccation.

Spore samples were prepared in sealed glass vials harboring a defined amount of dried *B*. *subtilis* spores (10^6^) with silica gel beads to maintain desiccation (**Fig 1**). To prepare the glass vials, 100 μL of *B*. *subtilis* spore stock solution with a concentration of 9.11 x 10^6^ CFU/mL was pipetted into sterile glass vials with gel loading pipette tips to reduce splash onto the vial walls. The vials were then dried down in silicon bead desiccators for 1–2 weeks before being sealed. To prepare the packages for the experiment, the neck of the vial was heated, the vial lid was pulled to seal the glass vials containing the desiccated spores, and the vials were put in their respective sample containers. Each time point consisted of two sets of three separate glass vials where each set was contained in a cardboard box. The cardboard box is located in a large oak box at the University of Edinburgh with an experimental duplicate at the Natural History Museum in London, UK. To perform the 500-year experiment, a set of vials is removed from the box every two years for the first 24 years and then every 25 years for the next 475 years (**S1 Fig**). To achieve this, sufficient sample vials to last the study timescale (500 years) were prepared, totaling 400 vials of spore samples, and the University of Edinburgh and the Natural History Museum will arrange both sample collection and analysis for each sampling time point. Logistics for when the sampling regimen decreases to once every 25 years have not yet been determined. Each sample box contains information on sampling interval and instructions in both written and electronic form. At each 25-year time point, the researchers must copy the instructions to ensure longevity and keep instructions updated with regard to technological and linguistic development. Because preservation is of the utmost importance, paper and ink of archival quality must be utilized. The first two time points (2014 and 2016) were recorded in this study. At each time point, a set of glass vials (n = 3) were opened and dried spores were recovered and tested for viability.

In addition to initiating the 500-year storage experiment, smaller time scale storage experiments were conducted to investigate the impact of various environmental agents on spore longevity and viability. Spore samples were stored in different stresses including -80°C, space-like vacuum, ambient air, and anoxic conditions as well as in different media including halite, simulated Martian regolith powder, and various aqueous NaCl solutions (0, 1.2 M, and 3.6 M). Conditions for all storage experiments are described in **Tables 1 and 2**. All spore samples started with 10^7^ spores, except for the aqueous NaCl incubations (pH of ~7), which started with 10^8^ spores. For cold (-80°C), ambient air, and anoxic conditions, samples were stored as air-dried spores sealed in glass vials. Those in -80°C were monitored over the course of 360 days. Ambient air samples contained a controlled atmosphere of 80% N_2_ and 20% O_2_ and temperature of 4°C and anoxic samples maintained an atmosphere of 99.9% CO_2_ at 4°C. Spore viability of ambient and anoxic air samples was monitored every 2 years for ten years.

**Table 1:** Conditions for storage of air-dried *B*. *subtilis* spores*.

Condition	Material additives	Amount of initial spores	Length of storage	Temperature (°C)	Atmospheric conditions
**500 year desiccation**	n.a.	10^6^	2 years	20 ± 3	Ambient air
**-80°C**	n.a.	10^7^	360 days	-80	Ambient air
**Vacuum**	n.a.	10^7^	450 days	20 ± 3	10^−7^ Pa pressure
**Controlled ambient air**	n.a.	10^7^	10 years	4	80% N_2_, 20% O_2_
**Anoxic**	n.a.	10^7^	10 years	4	99.9% CO_2_
**Simulated Mars regolith powder**	47.7% Na-montmorillonite, 9.9% kaolinite, 21.3% hematite, 13.0% anhydrite, 7.1% MgSO_4_, 1.0% NaCl, 2.5% Na_2_O, 3.4% MgO, 14.1% Al_2_O_3_, 34.6% SiO_2_, 5.1% SO_3_, 0.2% Cl^-^, 0.2% K_2_O, 6.1% CaO, 0.1% TiO, and 18.5% FeO	10^7^	10 years	4	Ambient air
**Halite powder**	99.5% NaCl	10^7^	10 years	4	Ambient air

*Unless otherwise noted, storage was performed in ambient air, 40 + /- 5% relative humidity, and normal laboratory conditions.

n.a. is defined as not-applicable

**Table 2:** Conditions for liquid storage of *B*. *subtilis* spores*.

Concentration of NaCl [M]	Amount of initial spores	Length of storage (weeks)
0	10^8^	52
1.2	10^8^	52
3.6	10^8^	52

*Storage was carried out in 4°C, ambient air.

In additional experiments spanning ten years, spores were stored in two different powders. In these experiments 10^7^ spores were to either (i) a powder composed of rock salt (halite containing 99.5% NaCl) or (ii) simulated Mars regolith powder containing a mineralogical composition according to the spectral data and information from the NASA Mars Exploration Rover *Spirit and Opportunity* [39] and OMEGA/Mars Express observations [40,41]. Spore-powder mixtures were air-dried, ground, and stored in reaction tubes at 4°C and monitored at two-year intervals for 10 years. For studying the effect of vacuum-induced extreme desiccation and space-like vacuum storage conditions, samples were exposed to ultrahigh vacuum produced by an ion-getter pumping system (400 liter/s; Varian SpA, Torino, Italy) that reached a final pressure of 10^−7^ Pa [42,43]. Samples remained at room temperature (20 ± 3°C) and were monitored over a period of 450 days. To assess spore viability over a gradient of NaCl concentrations, 10^8^ spores were stored in 10 ml solutions of 0 M, 1.2 M, and 3.6 M NaCl in H_2_O over 52 weeks and checked at regular intervals (**Table 2**).

### Live-cell imaging

To test whether individual spores recovered from the glass vials were capable of germination and outgrowth, spores were resuspended in the glass vial with 40 μL sterile water. A drop (7 μL) of this suspension was applied to a cell culture dish with a thin plastic bottom (μ-dish 35 mm, ibidi GmbH, Germany) and dried for 20 minutes at ambient room temperature. Then, spores were covered with a thin (~ 1 mm thickness) layer of 1.5% LB-agar to initiate germination and imaged at 37°C in a temperature-controlled incubation system by an automated inverted light microscope (TE2000-E Eclipse, Nikon) using phase-contrast and a NA of 1.3. Images were recorded with a digital color CCD camera (DS-2Mv, Nikon) at a resolution of 1600x1200 Pixel (12 bit) and with 5 seconds interval.

### Spore resistance treatments

For baseline data, spore samples from time point zero (2014) of the 500-year study were exposed to various environmental factors that can negatively affect long-term spore survival. Measurements of spore resistance to X-rays, 254 nm UV-C, 10% H_2_O_2_, dry heat (120°C) and wet heat (100°C) were performed on 10^7^ spores as previously described [16,32,44]. Ionizing radiation was administered to air dried spore samples in the form of X-rays (150 keV/19 mA) generated by an X-ray tube (Mueller type MG 150, MCN 165; Phillips, Hamburg, Germany) as described in Moeller et al. 2007 [44]. Dosimetry and dose calculations were performed as described previously [45]. Air-dried spore monolayers were exposed to monochromatic UV-C radiation from a low-pressure mercury lamp (NN 8/15; Heraeus, Berlin, Germany) with a major emission line at 254 nm. Heat (100°C) and oxidative (10% H_2_O_2_) stress were applied to spore samples in aqueous solution with 10^7^ spores/mL. To expose samples to dry heat, the air-dried spore samples in monolayers were incubated at 120°C.

### Recovery and evaluation of spore survival

Spores were recovered from glass vials by first sterilizing the vial exterior with ethanol and then placing a sterile Eppendorf microcentrifuge tube over the vial top to use as a lever to break the vial at the neck to open. 100 μL of buffered M9 medium was added to the vial and a small strip of ethanol sterilized parafilm was used to seal the vial. The vial was then was vortexed several times for short 10-second bursts for resuspension. With at least 10^6^ spores per sample, we expect the spores to be uniformly deposited in a monolayer fashion thereby decreasing the possibility of spore aggregation during rehydration, which could affect measurements of spore viability. Each vial (3 replicates for each time point) was checked for colony forming ability. Colony forming units (CFU) were checked by spreading 50 μl aliquots of serial dilutions in sterile water on nutrient broth (NB) agar plates. CFU were counted after 1 day at 37°C.

Spore samples containing powdered materials were directly resuspended in 10 mL sterile distilled water, vortexed and sonicated to guarantee spore separation as described in detail by Brown et al. 2007 [46]. Spores were recovered from all air-dried storage samples by covering the samples with sterile 10% aqueous polyvinyl alcohol (PVA) solution and dried [45,47]. The PVA layer was then removed aseptically as described previously [44,48] and resuspended in 1 mL sterile distilled water, resulting in >95% recovery of the spores. This procedure has no geno- or cytotoxic effects on spore viability [47]. Spore survival was determined by standard colony formation assays; resuspended spores and spores stored in NaCl solutions were serially diluted in sterile distilled water, aliquots applied to nutrient broth agar plates (Difco, Detroit, MI) and colonies counted after incubation overnight at 37°C, all as described previously [44,48,49].

### Numerical and statistical analysis

Spore survival was determined from the quotient N/N_0_, where N is the average CFU (colony forming units) of treated samples and N_0_ is the average CFU of untreated controls. The logarithm of N/N_0_ was plotted as a function of each treatment to obtain survival curves. Spore inactivation was expressed as the lethal dose at which 90% of the spore population is inactivated (LD_90_) [50]. Each experiment was performed in triplicate and all data are expressed as averages ± standard deviations. Significant differences in survival rates were calculated by single-factor analysis of variance (ANOVA), using SigmaPlot software Version 13.0 (Systat Software GmbH, Erkrath, Germany). Differences were considered statistically significant at P < 0.05.

## Results

**Fig 2:**
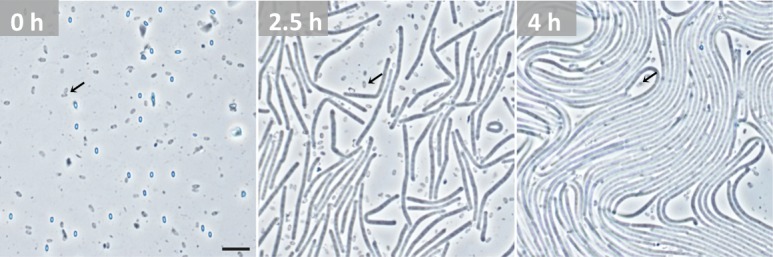
Live cell microscopy of germinating *B*. *subtilis* spores recovered from baseline samples in 500-year storage conditions. Images of three time points of germination are shown: 0, 2.5 and 4 h (see **S1 Movie** for an entire image sequence at 5 sec intervals). Note, that time point 0 h marks the beginning of the imaging. Activation of spores started 1–2 minutes before by adding a layer of LB-agar on top of the spores (see Materials & Methods section). A subpopulation of spores (~ 17%) was not capable of germination (arrows). Non-germinating spores appeared rather grey in phase-contrast without the bright core and darker ring-like boundary which is typical for dormant spores capable of germination (**see S2 Fig**). Scale bar = 5 μm.

The dried and enclosed *B*. *subtilis* spores were able to germinate at the beginning of the storage experiment (baseline) as revealed by cultivation experiments and by live cell microscopy (**Fig 2, S1 Movie**). After two years of storage, *B*. *subtilis* spores in the 500-year experiment exhibited no significant decrease in viability– 2016 samples had an averaged surviving fraction of 86 ± 21%.

When baseline (2014) *B*. *subtilis* spores were exposed to X-rays, UV-254 nm, 10% H_2_O_2_, wet heat and dry heat (**S3, S4, S5, S6 and S7 Figs**)), oxidative stress with 10% H_2_O_2_ was the most detrimental to spore populations with an LD_90_ value of just 9.54 ± 1.06 min (**Table 3**). Spores exposed to dry heat (120°C) showed greater resistance than those exposed to wet heat (100°C). In wet heat, spores reached 90% activation in one-fourth of the time (3.01 ± 0.39 min) compared to that of dry heat (14.75 ± 2.31 min), but dry heat resulted in more of a linear decrease in spore survival (**S5 Fig**). Radiation stress via X-ray and UV radiation was administered to air-dried spore monolayers and LD_90_ were 780.5 ± 62.4 Gy and 326.5 ± 29.5 J/m^2^, respectively. Resistance experiments were only conducted on 2014 baseline samples because 2016 desiccated spore samples did not yet show significant negative effects from storage. **Table 3** displays all LD_90_ values from this study as well as comparisons to previously published values.

**Table 3:** Resistance of *B*. *subtilis* baseline spores of the 500-year experiment to various agents*.

Treatment	Observed LD_90_	Published LD_90_	Reference
X-ray (Gy)	780.5 ± 62.4	838.1 ± 98.0	[44]
UV-254 nm (J/m^2^)	326.5 ± 29.5	273.1 ± 52.2	[44]
H_2_O_2_ (10%) (min)	9.54 ± 1.06	43.7 ± 1.9 ^a^	[50]
Wet heat, 100°C (min)	3.01 ± 0.39	17.4 ± 3.5 ^b^	[50]
Dry heat, 120°C (min)	14.75 ± 2.31	19 or 4.6 ± 0.2 ^c^	[16], [50]

*Baseline spores were treated with various agents, and spore survival was determined, all as described in Methods (**see S3–S7 Figs**).

^a^ Reported at 5% H_2_O_2_

^b^ Reported at 90°C

^c^ Reported at 100°C

**Fig 3:**
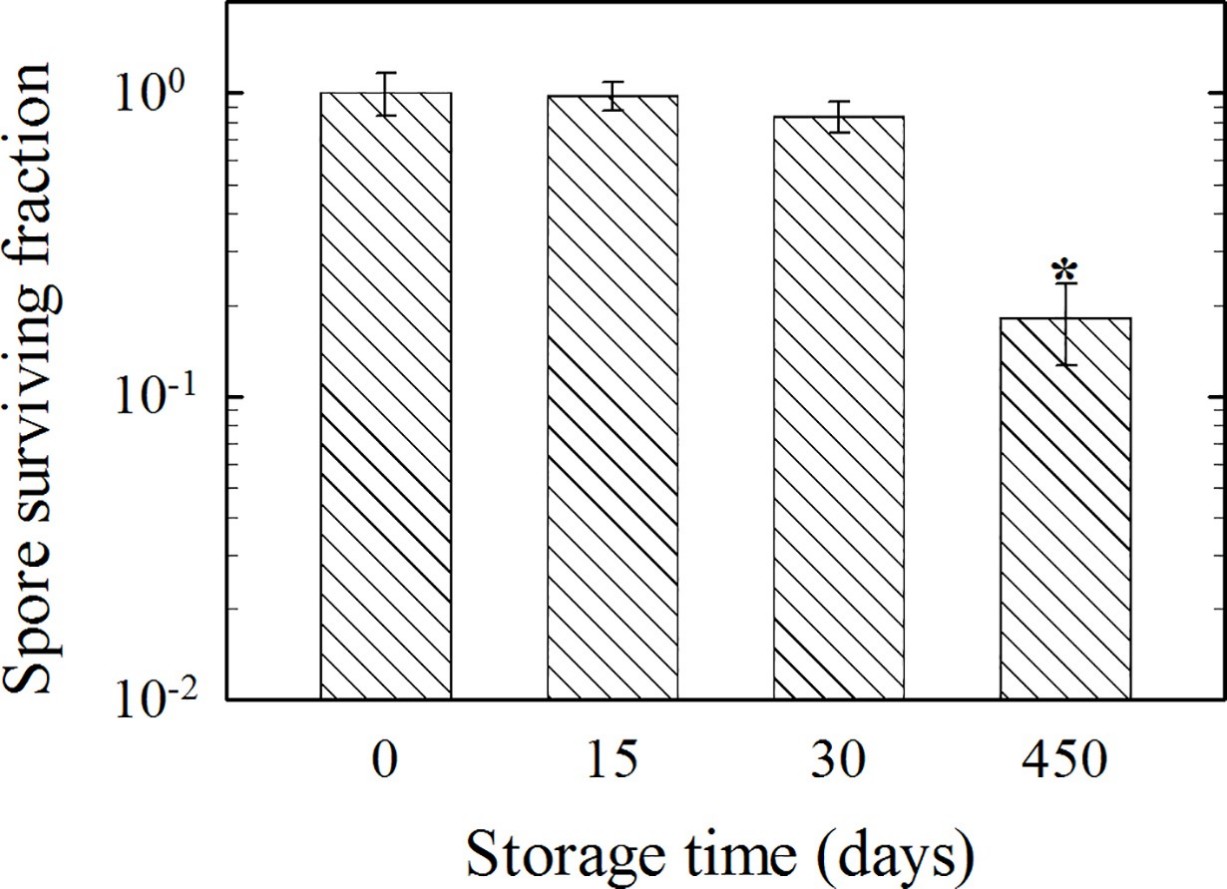
Spore survival during storage in space-like vacuum (10^−7^ Pa). Dry spores were stored at 10^−7^ Pa and spore survival was determined as described in Methods. Error bars represent standard deviation. Significance by ANOVA between different incubation times is denoted by (*) with p<0.001.

Ten-year storage experiments in dry conditions demonstrated that spore populations in ambient air (4°C), anoxic air (4°C), simulated Mars regolith powder and halite powder had no significant losses in spore viability (**Table 4**). Projections for LD_90_ were well over 300 years for each condition, ranging from 380.6 to 1789.7 years. Storage at -80°C for 360 days caused no significant decrease in *B*. *subtilis* spore viability–surviving fraction of 72.2 ± 11.8% after 360 days. However, when *B*. *subtilis* spores were stored for 450-days in ultrahigh vacuum (10^−7^ Pa), spore survival decreased by ~82% (p < 0.001) (**Fig 3**). The LD_90_ was estimated be to less than 2 years within these space-like conditions (**Table 4**).

**Table 4:** LD_90_ (90% spore inactivation) ranges for storage under different conditions*.

Treatment	Storage time	LD_90_ (years)^a^
**500-year desiccation**	2 years	29.5–45.3
**Halite**	10 years	699–829.4
**Mars regolith**		380.6–524.2
**Ambient air**		1616.9–1789.7
**Anoxic air**		414.3–506.9
**Vacuum (10^−7^ Pa)**	450 days	1.54–1.78
**0 M NaCl solution**	1 year	38.2–45.6
**1.2 M NaCl solution**		n.a.
**3.6 M NaCl solution**		3.1–3.7

*Spore storage experiments were carried out and spore survival measured all as described in Methods. The range was calculated using the standard deviation from the average of triplicate samples. However, the LD_90_ could not be calculated for 1.2 M NaCl solution storage because survival did not drop below 100%.

^a^ Data obtained by extrapolation and assuming log-linearity. However, it is not known whether log linearity will be maintained over the long storage times.

n.a. denotes an uncalculated LD_90_

**Fig 4:**
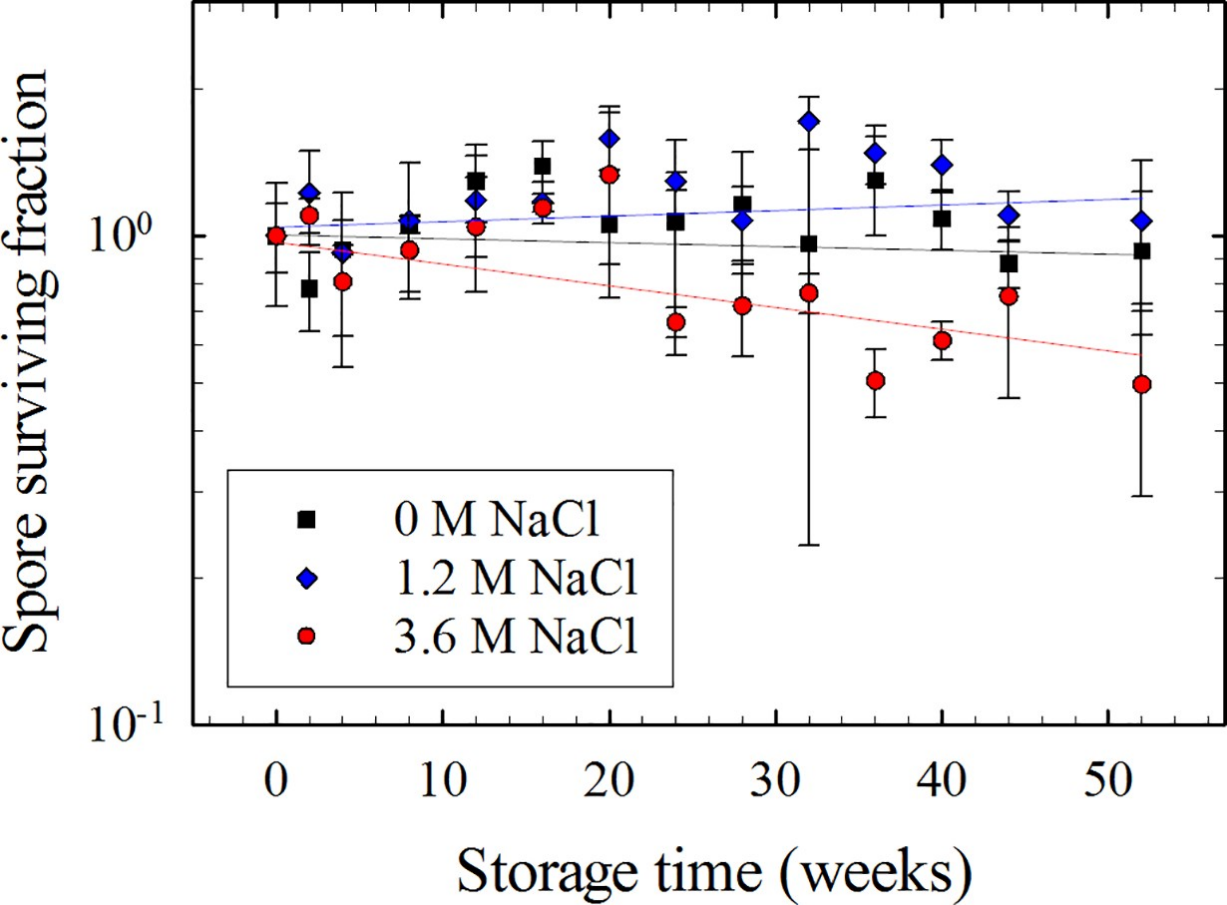
Spore survival in various NaCl solutions. Spores, 10^8^ per sample, were stored in solutions with various NaCl concentrations, and spore survival was measured as described in Methods. Black squares denote spores in water (0 M NaCl); blue diamonds denote spores in 1.2 M NaCl; and red circles denote spores in 3.6 M NaCl. Error bars signify the standard deviation.

*B*. *subtilis* spores stored in 3.6 M NaCl also exhibited a loss in viability over 1 year. While spore viability in 0 M and 1.2 M NaCl did not decrease, significant differences were observed in 3.6 M NaCl (**Fig 4 and S1 Table**). Total spore survival decreased by about ~50% in 3.6M NaCl after one year, with a projected LD_90_ ranging from 3.1–3.7 years (**Table 3**).

## Discussion

The pursuit of understanding the limits of life, in terms of both longevity and extreme resistance, is still ongoing. *B*. *subtilis* spores, in particular, are documented to tolerate many environmental extremes and are thus useful for understanding the fundamental mechanisms of dormancy, resistance and revival. This study will address the longevity of desiccated *B*. *subtilis* spores as well as that of the photosynthetic *Chroococcidiopsis sp*.–performed by the Cockell laboratory. Project details have been outlined previously in [36,37].

Following two years of storage in the conditions for the 500-year experiment, there was no significant loss in spore viability. However, prolonged storage in desiccation can be damaging to cell components by causing (i) conformational changes in the lipid membranes, (ii) cross-linking within carbohydrates, proteins, and nucleic acids, or (iii) polymerization of biomolecules, all of which can negatively affect cellular functions [51]. Previous research reports that DNA protection by α/β-type SASPs and efficient mechanisms for repair of DNA damage contribute to *B*. *subtilis* spores’ ability to remain viable following exposure to extreme dryness [12]. With a reduced water content of 38 ± 7% total weight of spores within the stored glass vials, proteins are also immobilized and enzymes inactivated, which may allow for prolonged dormancy [52]. The extent to which these factors can protect and stabilize DNA to allow long periods of survival in desiccation cannot be stated this early in our study. While there are no significant differences in survivability, minor differences may lead to big differences as the 500-year storage study progresses. *B*. *subtilis* spores have survived ten-year storage in various environments with no significant decrease in spore survival (**Table 4**), as well as nearly six years in space vacuum (1–2% survival in monolayers) as described by Horneck et al., [51]. Upon exposure to other extreme conditions including X-ray and UV radiation, wet heat and dry heat, baseline *B*. *subtilis* spore samples demonstrated similar LD_90_ when compared to previously published values (**Table 3**). However, as storage time increases, spore resistance to these conditions may be dramatically affected.

*B*. *subtilis* spore viability appeared unaffected by storage in artificial settings such as Mars regolith and halite powders (**Table 4**). Conversely, storage in aqueous solutions with elevated salinity showed a significant decrease in spore survival as compared to storage in NaCl powder, suggesting that *B*. *subtilis* spores are particularly susceptible to very concentrated NaCl solutions. The detrimental effects of salinity on microbial communities [53] and specifically on *B*. *subtilis* vegetative cells [54] are well characterized. Further, recent studies indicate that high salinity can be inhibitory for *B*. *subtilis* spore germination by causing a delay in germination onset [55,56]. The LD_90_ for spores stored in 3.6 M NaCl was estimated at 3.4 ± 0.3 years, which is significantly less time when compared to those in halite powder (764.2 ± 65.2 years). Nonetheless, it reveals that *B*. *subtilis* spores in the short-term storage have a marginal osmoresistance, a quality that has been documented for various *Bacillus* spores [57].

Altogether, we hope that our study will shed light on whether spores of at least one species can realistically survive for centuries in desiccation and whether the potential compounding damage from prolonged desiccation can be combatted. Because this study is set for an extended timescale, it should be noted that spontaneous germination may occur, albeit at an extremely low frequency [58,59]. However, we expect the degree of desiccation within the glass vials to substantially reduce the likelihood of spontaneous germination of the stored spores. Importantly, with 498 more years left of this study, there is also room for project development as our capabilities for investigation advance. For example, it may become possible to sequence low amounts of nucleic acids from germinated (viable) recovered spores to ascertain what changes and mutations, if any, are occurring at the genome level as storage time increases e.g. every 25 years. In doing so, we can systematically quantify the way in which these spores die over extended timescales and understand the pathways and failures lying therein. This study may have associated implications of re-activating a microorganism that has potentially survived half a millennium of dormancy. Nevertheless, this experiment will provide valuable samples and project possibilities for the future. There is a great deal of time left for data collection, documentation and study development.

## Supporting information

S1 Fig Schematic of 500-year study sampling timeline. For the first 24 years (stage 1), spore viability tests will be performed every 2 years. For the remaining 475 years (stage 2), sampling will decrease to once every 25 years. Each sampling point is denoted by a vertical line. Dark blue signifies what data has been collected; light blue signifies data to be collected as the study continues.
(TIF)

S2 Fig Morphology of *B*. *subtilis* spores from 500-year storage baseline samples. Samples were fixed with 2.5% glutaraldehyde in HEPES buffer. A) Phase contrast light microscopy showed that spores with two morphologies are present in the baseline sample. Besides dormant spores, which showed the typical compressed ring-like morphology, grey or black spores could be detected (red arrows). Live-cell imaging demonstrated that these atypical spores did not germinate (see **Fig 2**). B) Scanning electron microscopy (SEM) showed that some of the spores (yellow arrow) possessed a collapsed shape and an unusual surface structure (i.e. the rucks of the coat are missing). Scale bar in A = 5 μm and in B = 200 nm.
(TIF)

S3 Fig *B*. *subtilis* spore resistance to X-rays. The experiment was performed with baseline 500-yr storage samples in triplicate as described Methods with error bars representing the standard deviation from the average (n = 3). The loss in spore viability follows a linear (1^st^ order) function with r^2^ = 0.9924.
(TIF)

S4 Fig *B*. *subtilis* spore resistance to 254 nm UV-C radiation. The experiment was performed with baseline 500-yr storage samples in triplicate as described Methods with error bars representing the standard deviation from the average (n = 3). The loss in spore viability follows a 1^st^ order function with r^2^ = 0.9817.
(TIF)

S5 Fig *B*. *subtilis* spore resistance to dry heat (120°C). The experiment was performed with baseline 500-yr storage samples in triplicate as described Methods with error bars representing the standard deviation from the average (n = 3). The loss in spore viability follows a 1^st^ order function with r^2^ = 0.9960.
(TIF)

S6 Fig *B*. *subtilis* spore resistance to oxidative stress (10% H_2_O_2_). The experiment was performed with baseline 500-yr storage samples in triplicate as described Methods with error bars representing the standard deviation from the average (n = 3). The loss in spore viability follows a 1^st^ order function with r^2^ = 0.9957.
(TIF)

S7 Fig *B*. *subtilis* spore resistance to wet heat (100°C). The experiment was performed with baseline 500-yr storage samples in triplicate as described Methods with error bars representing the standard deviation from the average (n = 3). The loss in spore viability follows a 2^nd^ order function with r^2^ = 0.9441.
(TIF)

S1 Table Spore survival in various NaCl solutions over a year. (DOCX)

S1 Movie Live cell imaging of germinating *B*. *subtilis* spores. Spores were deposited on ibidi-dishes from baseline 500-year storage glass vial samples, covered by LB-agar and imaged by phase-contrast microscopy at 5 sec intervals. Spores with the typical morphology (bright core with a dark ring-like boundary) germinated (switch from phase-bright to phase-dark core) and grew out. In contrast, grey spores did not change in their morphology (see also **S2 Fig**).
(AVI)
