# Supplementary figures and images for: Experimental studies addressing the longevity of Bacillus subtilis spores – The first data from a 500-year experiment
Source: PLoS One. 2018 Dec 4;13(12):e0208425. doi: 10.1371/journal.pone.0208425 (PMC6279046; doi:10.1371/journal.pone.0208425)

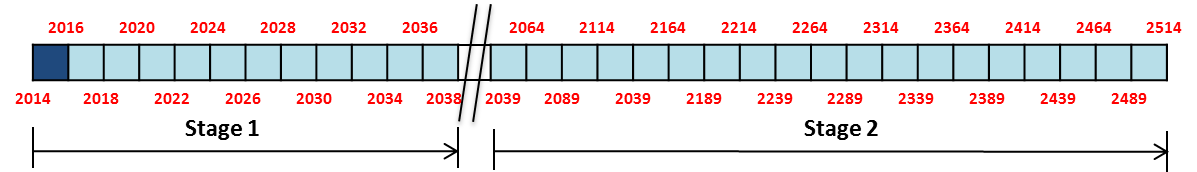

Supplement: S1 Fig — For the first 24 years (stage 1), spore viability tests will be performed every 2 years. For the remaining 475 years (stage 2), sampling will decrease to once every 25 years. Each sampling point is denoted by a vertical line. Dark blue signifies what data has been collected; light blue signifies data to be collected as the study continues. (TIF) [file pone.0208425.s001.tif]

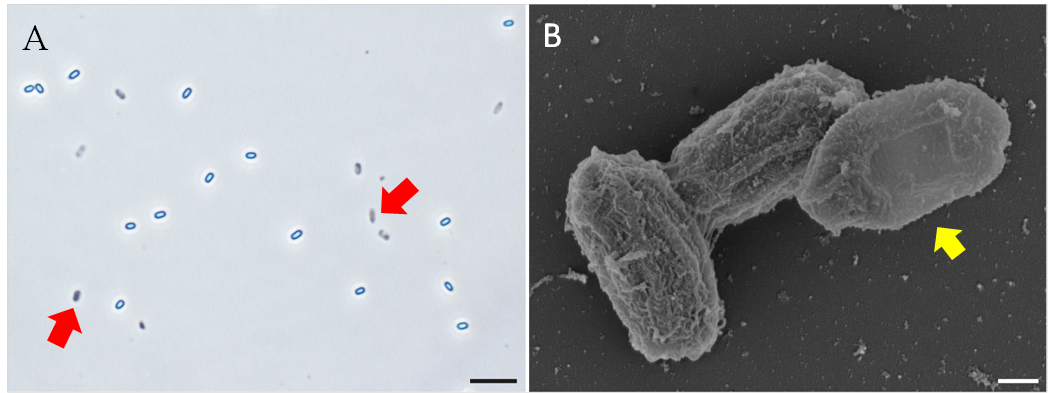

Supplement: S2 Fig — Samples were fixed with 2.5% glutaraldehyde in HEPES buffer. A) Phase contrast light microscopy showed that spores with two morphologies are present in the baseline sample. Besides dormant spores, which showed the typical compressed ring-like morphology, grey or black spores could be detected (red arrows). Live-cell imaging demonstrated that these atypical spores did not germinate (see Fig 2). B) Scanning electron microscopy (SEM) showed that some of the spores (yellow arrow) possessed a collapsed shape and an unusual surface structure (i.e. the rucks of the coat are missing). Scale bar in A = 5 μm and in B = 200 nm. (TIF) [file pone.0208425.s002.tif]

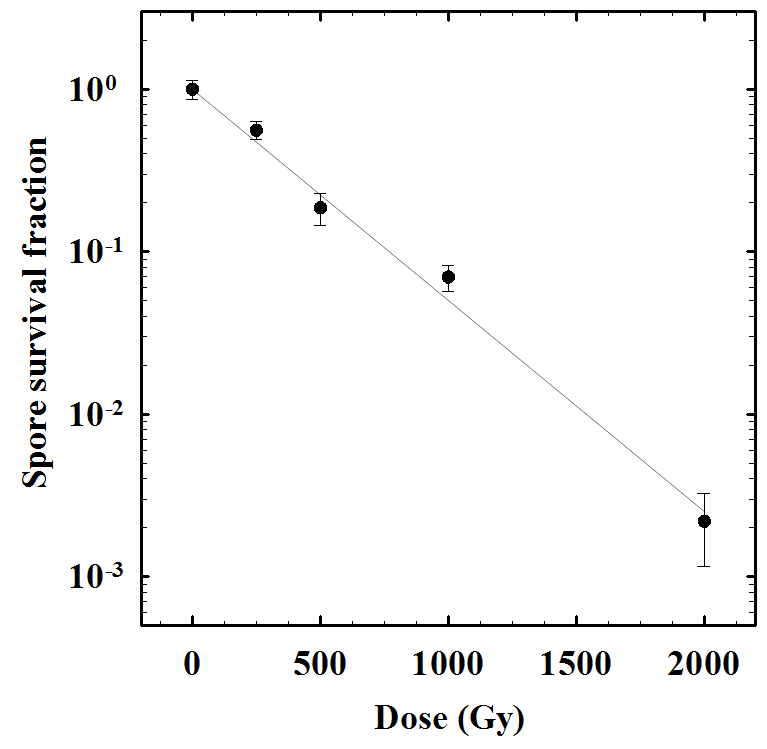

Supplement: S3 Fig — The experiment was performed with baseline 500-yr storage samples in triplicate as described Methods with error bars representing the standard deviation from the average (n = 3). The loss in spore viability follows a linear (1st order) function with r2 = 0.9924. (TIF) [file pone.0208425.s003.tif]

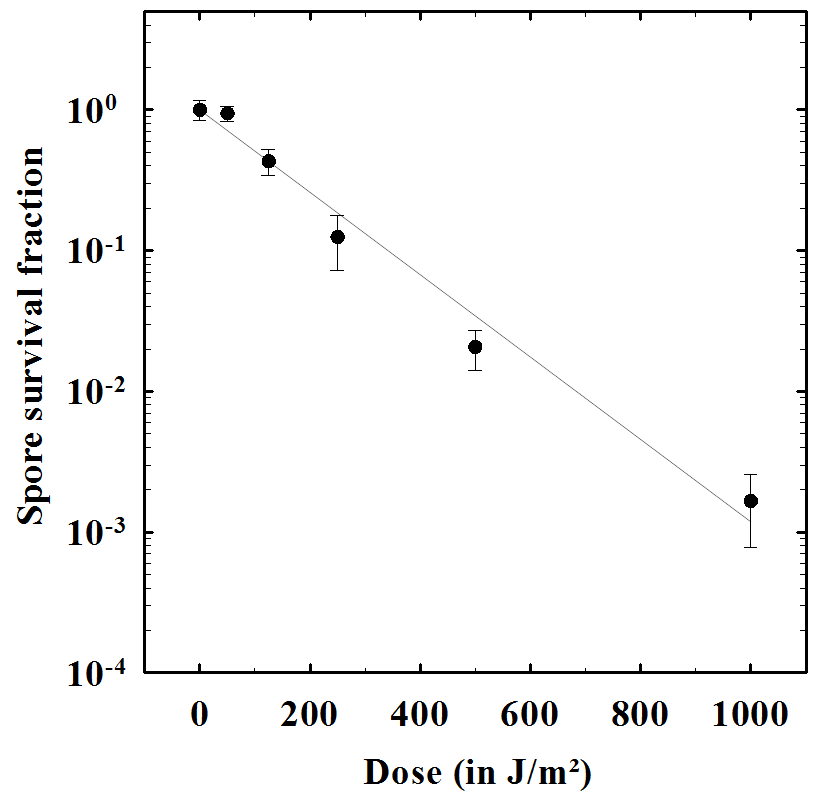

Supplement: S4 Fig — The experiment was performed with baseline 500-yr storage samples in triplicate as described Methods with error bars representing the standard deviation from the average (n = 3). The loss in spore viability follows a 1st order function with r2 = 0.9817. (TIF) [file pone.0208425.s004.tif]

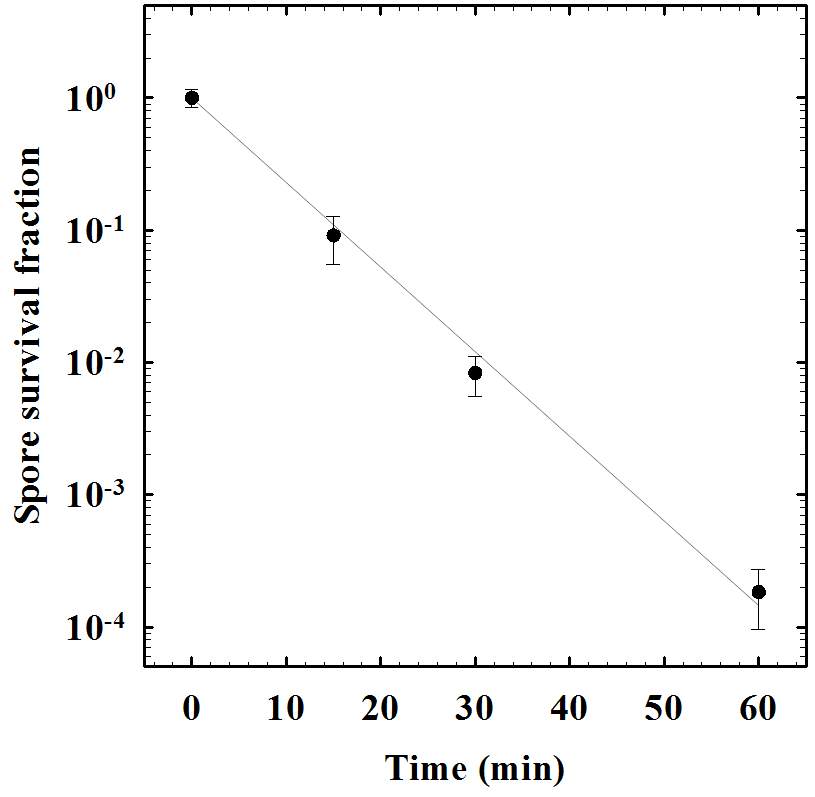

Supplement: S5 Fig — The experiment was performed with baseline 500-yr storage samples in triplicate as described Methods with error bars representing the standard deviation from the average (n = 3). The loss in spore viability follows a 1st order function with r2 = 0.9960. (TIF) [file pone.0208425.s005.tif]

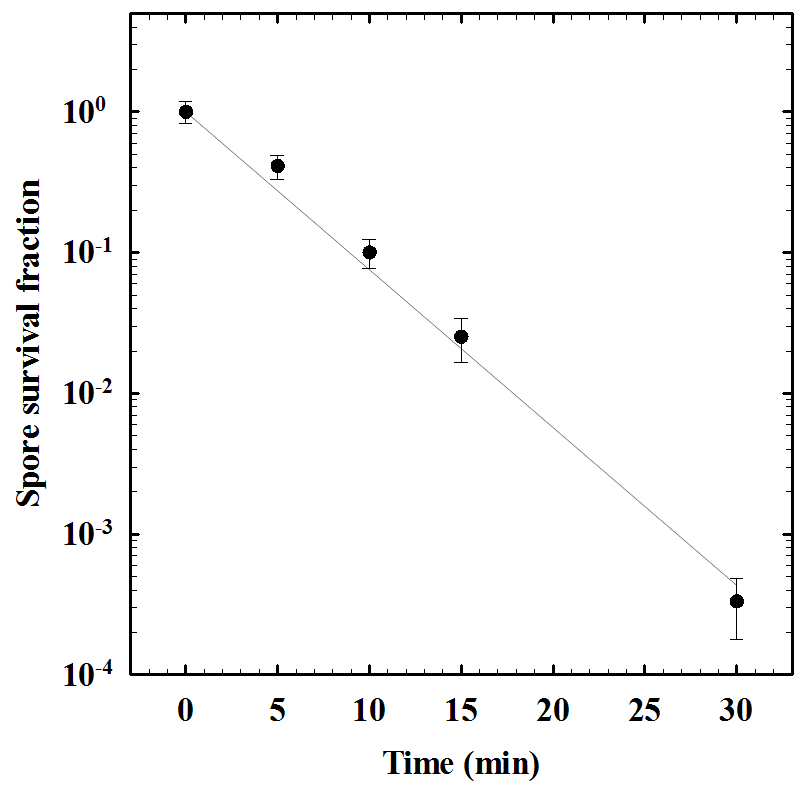

Supplement: S6 Fig — The experiment was performed with baseline 500-yr storage samples in triplicate as described Methods with error bars representing the standard deviation from the average (n = 3). The loss in spore viability follows a 1st order function with r2 = 0.9957. (TIF) [file pone.0208425.s006.tif]

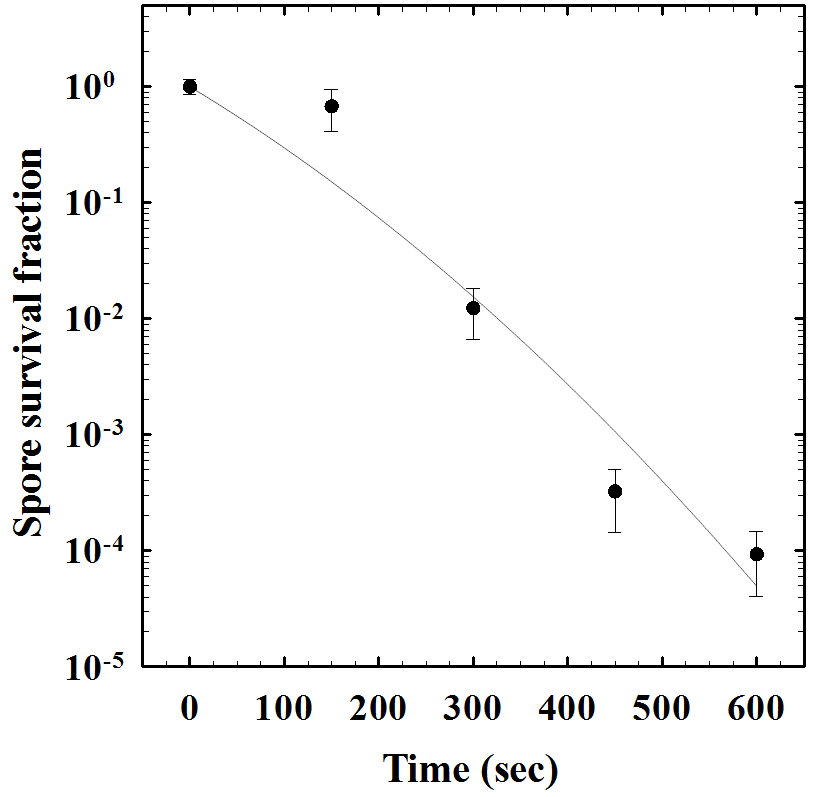

Supplement: S7 Fig — The experiment was performed with baseline 500-yr storage samples in triplicate as described Methods with error bars representing the standard deviation from the average (n = 3). The loss in spore viability follows a 2nd order function with r2 = 0.9441. (TIF) [file pone.0208425.s007.tif]
