# Supplementary material for: Experimental studies addressing the longevity of Bacillus subtilis spores – The first data from a 500-year experiment
Source: PLoS One. 2018 Dec 4;13(12):e0208425. doi: 10.1371/journal.pone.0208425 (PMC6279046; doi:10.1371/journal.pone.0208425)
Supplement: S1 Table — (DOCX) [file pone.0208425.s008.docx]

**S1 Table. Spore survival in various NaCl solutions over a year.***

|  | Spore survival (average ± standard deviation) | | |
| --- | --- | --- | --- |
| **Storage time (weeks)** | **0 M NaCl** | **1.2 M NaCl** | **3.6 M NaCl** |
| 0 | 1.00 ± 0.28 | 1.00 ± 0.16 | 1.00 ± 0.16 |
| 2 | 0.78 ± 0.14 | 1.22 ± 0.27 | 1.10 ± 0.09 |
| 4 | 0.93 ± 0.02 | 0.92 ± 0.30 | 0.81 ± 0.27 |
| 8 | 1.05 ± 0.04 | 1.07 ± 0.33 | 0.94 ± 0.17 |
| 12 | 1.30 ± 0.23 | 1.18 ± 0.28 | 1.04 ± 0.27 |
| 16 | 1.39 ± 0.17 | 1.17 ± 0.11 | 1.14 ± 0.02 |
| 20 | 1.06 ± 0.31 | 1.58 ± 0.25 | 1.33 ± 0.46 |
| 24 | 1.07 ± 0.50 | 1.29 ± 0.06 | 0.67 ± 0.05 |
| 28 | 1.16 ± 0.32 | 1.07 ± 0.19 | 0.72 ± 0.15 |
| 32 | 0.96 ± 0.73 | 1.71 ± 0.21 | 0.76 ± 0.07 |
| 36 | 1.30 ± 0.30 | 1.48 ± 0.20 | 0.51 ± 0.08 |
| 40 | 1.08 ± 0.15 | 1.40 ± 0.17 | 0.61 ± 0.06 |
| 44 | 0.88 ± 0.10 | 1.10 ± 0.13 | 0.75 ± 0.29 |
| 52 | 0.93 ± 0.30 | 1.07 ± 0.35 | 0.50 ± 0.20 |

*****Baseline spores were incubated in solutions of various NaCl concentration, and spore survival was determined at various times, all as described in Methods.
